# Medical learning in a private hospital: patients’ and companions’ perspectives

**DOI:** 10.1590/S1516-31802009000200009

**Published:** 2009-07-06

**Authors:** Arthur de Carvalho Jatobá e Sousa, Carolina da Rocha Machado Tajra, Rodolfo de Souza Coelho, Ciro Martins Gomes, Ricardo Afonso Teixeira

**Affiliations:** 1 Medical student at the Universidade Católica de Brasília (UCB), Brasília, Distrito Federal, Brazil.; 2 MD. Physician and former medical student at the Universidade Católica de Brasília (UCB), Brasília, Distrito Federal, Brazil.; 3 MD, PhD. Professor at the Universidade Católica de Brasília (UCB), Brasília, Distrito Federal, Brazil.

**Keywords:** Students, medical, Education, medical, Hospitals, private, Patient satisfaction, Questionnaires, Estudantes de medicina, Educação médica, Hospitais privados, Satisfação do paciente, Questionários

## Abstract

**CONTEXT AND OBJECTIVE:**

Contact with patients has important implications for medical students’ education. Previous studies have shown that patients in teaching hospitals have positive views about medical education. The aim here was to assess the acceptability of medical education among patients and their companions in a non-teaching private hospital that is planning to implement a medical teaching program in the near future.

**DESIGN AND SETTING:**

Cross-sectional study conducted in a 200-bed tertiary-care private hospital in Brasília.

**METHODS:**

Between March and April 2005, patients and their companions in three different sections of the hospital (intensive care unit, ward and emergency waiting room) were surveyed using a questionnaire.

**RESULTS:**

The questionnaire was completed by 209 volunteers. The majority of the volunteers (178; 85%) said that they would allow a student to be present during consultations. Of these, 102 (57%) said that they would like to have a student present. Acceptance of the presence of students was higher among males (males 93%; females 81%; P = 0.026). Intensive care unit respondents said that they would like medical students to be present more frequently than the other two groups said this (ward 48%; emergency room 49%; intensive care unit 74%; P = 0.011).

**CONCLUSIONS:**

Not only were medical students well accepted but also their presence during consultations was desired by many patients and their companions. These findings may be of great value for plans to implement medical teaching programs in private hospitals.

## RESUMO

**CONTEXTO E OBJETIVO:**

O contato com pacientes tem implicações importantes na educação de estudantes de medicina. Estudos prévios revelam que pacientes em hospitais escola têm opiniões positivas sobre ensino médico. O objetivo foi avaliar a aceitação de ensino médico em um hospital privado não-universitário que planeja implementar um programa de ensino médico em futuro próximo.

**TIPO DE ESTUDO E LOCAL:**

Estudo transversal conduzido em hospital privado de 200 leitos, em Brasília.

**MÉTODOS:**

Em Brasília entre março e abril de 2005, foram aplicados questionários a pacientes e acompanhantes em três diferentes seções do hospital: unidade de terapia intensiva, enfermaria e sala de espera do pronto-socorro.

**RESULTADOS:**

O questionário foi respondido por 209 voluntários. A maioria, 178 (85%), permitiria a presença do estudante de medicina durante a consulta e dentre estes, 102 (57%) gostariam da presença do estudante. A aceitação à presença de estudantes foi maior em voluntários do sexo masculino (homens 93%; mulheres 81%; P = 0,026). Os voluntários da unidade de terapia intensiva foram os que mais gostariam da presença do estudante quando comparados aos outros dois grupos (enfermaria 48%; pronto-socorro 49%; unidade de terapia intensiva 74%; P = 0,011).

**CONCLUSÕES:**

Estudantes de medicina são não apenas bem aceitos, mas também desejados por muitos pacientes e acompanhantes durante consultas médicas. Esses achados podem ter grande valor para o plano de implementação de um programa de ensino médico em um hospital privado.

## INTRODUCTION

Contact with patients has important implications for medical students’ education. Such contact enables students to develop professional skills and attitudes that will be of great value in their medical careers. Most studies on medical students’ acceptability among patients involve teaching settings.^1^^,^^2^^,^^3^

Patient satisfaction with care, which is a very important issue, seems not to be affected by the presence of medical students during consultations.^1^ Studies have shown that over 95% of patients have positive or neutral attitudes towards the presence of medical students.^2^^,^^3^ However, acceptance of the presence of students seems to decline if patients’ complaints concern emotional or sexual problems, or when an internal examination is required.^2^

## OBJECTIVE

The aim of our study was to assess the acceptability of medical education among patients and their companions in a non-teaching private hospital that is planning to implement a medical teaching program in the near future.

## MATERIAL AND METHODS

We carried out a cross-sectional observational study in a 200-bed tertiary-care private hospital in the city of Brasília, during March and April 2005. The study was conducted with full approval from the institutional review board and ethics committee. A questionnaire was passed out to three different groups: patients in the hospital ward, patients in the emergency waiting room and members of the families of patients admitted to the intensive care unit who were waiting for visiting hours.

The survey questionnaire was handed out by three members of the hospital’s customer service team, based on obtaining oral informed consent from volunteers. All the volunteers were given a standardized explanation about the purpose and importance of the survey that was being conducted. Volunteers were included in the study if they were more than 16 years old, were physically able to fill out the questionnaire and were willing to participate. All questionnaires were passed out to consecutive volunteers.

The survey questionnaire consisted of 16 questions divided into two main sections. The first section contained questions seeking demographic information and the second section contained questions seeking the volunteers’ attitudes towards the presence of students during medical consultations (Appendix 1). All questionnaires were filled out by the volunteers themselves, which took approximately two to five minutes.

We used the chi-squared test for statistical analyses and the significance level was set at 0.05. The sample size was calculated using the G-Power program.^4^ Assuming a median effect of 0.3, power of 95%, alpha of 0.05 and four degrees of freedom, the required sample size was 207.

## RESULTS

Two hundred and nineteen volunteers were approached by the hospital staff and invited to answer the survey questionnaire. All of the ward patients (75) and the family members of the intensive care unit patients (70) who were approached completed the questionnaire. Out of 74 patients approached in the emergency waiting room, 64 (86%) completed the survey. Some of the patients who did not want to participate said that they were not feeling well and others that they would soon be called in for their consultations.

The respondents’ mean age was 38.4 years (range 17-95); 142 (68%) were female, 93 (44%) were university graduates, 87 (42%) had monthly family income of more than 10 minimum salaries and 180 (86%) had health insurance. Their demographic characteristics are shown in Table 1.

One hundred and seventy-eight volunteers (85%) said that they would allow a medical student to be present during their consultations. Of these, 102 (57%) said that they would like to have a medical student present during their consultations and 68 (38%) were neutral to this question. In addition, acceptance of students was higher among males (males 93%; females 81%; P = 0.026).

We found no significant association between allowing the presence of a medical student and respondent age or family income. However, respondents who answered that they would like to have a medical student present during consultation had significantly lower monthly family income (P = 0.029).

**Figure 1.**
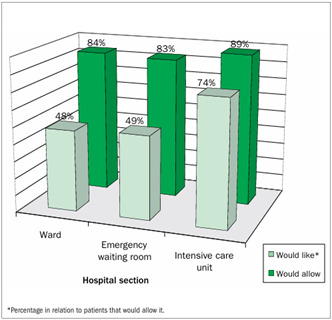
Percentages of respondents that would allow and would like the presence of a medical student during consultations.

Relatives of patients who were in the intensive care unit, compared with the other two groups, were the ones who said that they would like the presence of a medical student the most (ward 48%; emergency waiting room 49%; intensive care unit 74%; P = 0.011) (Figure 1).

Out of the 178 respondents who would allow the presence of a medical student, 102 (57%) believed that they would have more attention and better care if students were present and 123 (69%) would feel more comfortable if a medical resident was present instead of a medical student. The responses to the survey questions are shown in Table 2.

**Table 1.** Demographic characteristics

	Ward (n = 75)	ER (n = 64)	ICU (n = 70)	Total (n =209)
Men, n (%)	22 (29)	16 (25)	22 (31)	60 (29)
Women, n (%)	51 (68)	46 (72)	45 (64)	142 (68)
No response, n (%)	2 (3)	2 (3)	3 (4)	7 (3)
Age, mean (SD) [range]	41.3 (19.2) [17-95]	34.9 (12.1) [17-65]	38.4 (13.5) [18-69]	38.4 (15.6) [17-95]
Educational level, n (%)				
Some elementary school	4 (5)	2 (3)	3 (4)	9 (4)
Elementary school completed	5 (7)	1 (2)	3 (4)	9 (4)
Some high school	4 (5)	5 (8)	5 (7)	14 (7)
High school completed	11 (15)	18 (28)	9 (13)	38 (18)
Some university-level	24 (32)	9 (14)	11 (16)	44 (21)
University-level completed	26 (35)	28 (44)	39 (56)	93 (44)
No response	1 (1)	1 (2)	0 (0)	2 (1)
Brazilian minimum salaries, n (%)				
< 5	17 (23)	13 (20)	15 (21)	45 (22)
5 to 10	26 (35)	17 (27)	22 (31)	65 (31)
11 to 20	9 (12)	16 (25)	15 (21)	40 (19)
> 20	18 (24)	14 (22)	15 (21)	47 (22)
No response	5 (7)	4 (6)	3 (4)	12 (6)
Health insurance, n (%)				
Yes	70 (93)	53 (83)	57 (81)	180 (86)
No	4 (5)	9 (14)	13 (19)	26 (12)
No response	1 (1)	2 (3)	0 (0)	3 (1)

The percentages may not add up to 100%, due to rounding; ER = emergency room; ICU = intensive care unit; SD = standard deviation.

**Table 2.** Responses to survey questions on the presence of a medical student during consultations

Survey topics: respondents’ views	Ward (n = 63)	ER (n = 53)	ICU (n = 62)	Total (n = 178)
Would like to have a medical student present during consultation	30 (48%)	26 (49%)	46 (74%)	102 (57%)
Would feel more comfortable with up to two students	53 (84%)	37 (70%)	47 (76%)	137 (77%)
Believe that they would have more attention and better care	35 (56%)	25 (47%)	42 (68%)	102 (57%)
Believe that they would learn more and have a better understanding of their problem	41 (65%)	30 (57%)	41 (66%)	112 (63%)
Believe that it is important to have time alone with the doctor	49 (78%)	41 (77%)	47 (76%)	137 (77%)
Would not feel uncomfortable with a student of the opposite sex	51 (81%)	34 (64%)	53 (85%)	138 (78%)
Would allow students to see their medical charts	59 (94%)	36 (68%)	53 (85%)	148 (83%)
Consider it essential for the doctor to be present at all times during the consultation	53 (84%)	38 (72%)	53 (85%)	144 (81%)
Would feel more comfortable with a medical resident instead of a medical student	42 (67%)	33 (62%)	48 (77%)	123 (69%)

The number of respondents varied for some questions, due to non-response; ER = emergency room; ICU = intensive care unit.

## DISCUSSION

The magnitude of acceptance of medical students in our study is comparable to previous studies conducted in teaching settings.^2^^,^^3^ Apparently, patients and their companions see advantages in being involved in medical education. It seems that they see students as a means of receiving better care.

Other studies have suggested that the presence of medical students during consultations has many advantages for patients, including improved and more thorough consultation and better understanding of their problems.^1^^,^^3^ Physicians seem to benefit as well. They report greater enjoyment of the practice of medicine and increased time spent reviewing clinical medicine when they are involved in teaching medical students.^5^ Moreover, the quality of care is better in teaching hospitals than in non-teaching hospitals.^6^ Thus, a teaching environment appears to provide benefits for all parties involved.

We found that the acceptance of students’ presence was higher among males. Another study reported that females are less likely to wish to see a student for intimate problems.^2^ Males may be less self-conscious than females, not caring much whether students are present or not. A recent study^7^ reported that over 90% of patients were satisfied with consultations conducted by medical students and that most would consult with a student again. However, emotional problems and intimate examinations were matters for concern.

One limitation of our study was that the data from the intensive care unit was obtained from members of patients’ families, and not from the patients themselves. This could definitely be a factor influencing the higher acceptability of medical students in this group, compared with the other two groups. Another point to consider is that patients in the intensive care unit are much more debilitated and vulnerable than patients in the hospital ward or the emergency waiting room. Therefore, family members might see the presence of medical students as an opportunity to have better care and more information about their loved ones.

It is important to emphasize that although the majority of patients are willing to participate in medical education, they should always be given the choice of whether or not to participate. In addition, they should be informed that their care will not be affected in any way if they decide not to participate.

## CONCLUSION

Our study demonstrated a high level of acceptance of students in a non-teaching private hospital. This finding may be of great value for plans to implement medical teaching programs in private hospitals.
